# Lack of Transmission of Chronic Wasting Disease Prions to Human Cerebral Organoids

**DOI:** 10.3201/eid3006.231568

**Published:** 2024-06

**Authors:** Bradley R. Groveman, Katie Williams, Brent Race, Simote Foliaki, Tina Thomas, Andrew G. Hughson, Ryan O. Walters, Wenquan Zou, Cathryn L. Haigh

**Affiliations:** Rocky Mountain Laboratories, National Institute of Allergy and Infectious Diseases, Hamilton, Montana, USA (B.R. Groveman, K. Williams, B. Race, S. Foliaki, T. Tomas, A.G. Hughson, R.O. Walters, C.L. Haigh);; Jiangxi Academy of Clinical Medical Sciences, The First Affiliated Hospital of Nanchang University, Nanchang, China (W. Zou)

**Keywords:** prions, PrP, chronic wasting disease, CWD, human cerebral organoid, zoonoses, United States, China

## Abstract

Chronic wasting disease (CWD) is a cervid prion disease with unknown zoonotic potential that might pose a risk to humans who are exposed. To assess the potential of CWD to infect human neural tissue, we used human cerebral organoids with 2 different prion genotypes, 1 of which has previously been associated with susceptibility to zoonotic prion disease. We exposed organoids from both genotypes to high concentrations of CWD inocula from 3 different sources for 7 days, then screened for infection periodically for up to 180 days. No de novo CWD propagation or deposition of protease-resistant forms of human prions was evident in CWD-exposed organoids. Some persistence of the original inoculum was detected, which was equivalent in prion gene knockout organoids and thus not attributable to human prion propagation. Overall, the unsuccessful propagation of CWD in cerebral organoids supports a strong species barrier to transmission of CWD prions to humans.

Chronic wasting disease (CWD) is a member of the prion family of fatal, infectious neurodegenerative diseases. CWD affects cervids, such as moose, elk, and several species of deer, across much of North America, South Korea, and certain countries in northern Europe, including Norway, Finland, and Sweden (*1*). CWD is the most transmissible of the prion disease family; transmission between cervids is highly efficient. Another member of the prion disease family, bovine spongiform encephalopathy (BSE), has transmitted to humans and caused the emergence of variant Creutzfeldt-Jakob disease (vCJD). That transmission is widely believed to have occurred through ingestion of contaminated food. Subsequently, concern is ongoing as to whether CWD prions could likewise infect humans because of the high likelihood of CWD-tainted meat entering the human food chain.

Prions are formed by the conversion of the normal cellular prion protein (PrP) into abnormally folded isoforms. The current understanding is that, once formed, prions continue to propagate themselves by recruiting normally folded PrP molecules, which then undergo templated conversion into new prions (*2*,*3*). In humans, the amino acid sequence of PrP influences disease susceptibility, manifestation, and clinical course. A single amino acid polymorphism at codon 129 has been shown to influence susceptibility to prion disease, disease duration, phenotype, and the propensity of PrP to form amyloid (*4*,*5*). Persons can be homozygous for methionine (MM) or valine (VV) at codon 129 or heterozygous (MV), with the prevalence of each allele varying by geographic location (*5*). Worldwide, the 129 MM and MV genotypes comprise ≈80%–100% of the population (*5*). When BSE crossed the species barrier and transmitted to humans, the MM polymorphism was present in most symptomatic patients; only 1 possible and 1 confirmed case in patients carrying the MV polymorphism were identified (*6*–*8*). After the BSE epidemic, histological screening of appendices was used to attempt an approximation of the prevalence of preclinical or subclinical vCJD. This screening found abnormal PrP in all 3 genotypes in the ratio of 2:1:1 (MM:MV:VV), which indicated BSE infection might be possible in persons of all 3 genotypes (*9*). That finding is, however, confounded by the finding of the follow-up appendix-3 survey that abnormal PrP was present in 2 appendices removed before the known BSE exposure period (*10*). Therefore, although the onset of BSE exposures might have begun sooner than originally realized, resulting in earlier silent infections, a low background of abnormal (but not necessarily pathogenic) PrP could possibly be present in lymphoid tissue of the wider population, independent of BSE exposure (*10*). Despite this possibility, most patients who contracted vCJD from BSE-infected meat had methionine homozygosity at codon 129, supporting increased susceptibility of this genotype to infection.

In attempts to ascertain the risk to humans posed by CWD-tainted meat or other cervid-derived products, various studies have looked at the propensity of cervid prions to cross the species barrier, seed the conversion of human prions, and by inference determine the likelihood of causing human disease. In vitro studies have demonstrated that CWD prions can seed human PrP substrates (*11*,*12*), although conversion of human PrP was less efficient than for cervid PrP (*12*). Transmission studies in mice have principally shown that CWD prions do not readily infect transgenic mice expressing either normal or very high levels of human PrP (*13*–*17*). However, using the highly sensitive real-time quaking-induced conversion (RT-QuIC) assay, low levels of PrP seeding activity could be detected in 4/50 *tg66* mice inoculated with either elk or whitetail deer CWD, in the absence of any other indicators of disease (*18*). The *tg66* mice express levels of human PrP 8–16-fold above normal; when the same inocula were tested in mice expressing only 2–4-fold higher levels, no seeding activity was detected. Follow-up experiments passaging brain material from those 4 mice demonstrated a lack of prion infectivity in the brain, suggesting that what was detected was likely residual inoculum, or false-positive reactions, and not transmissible disease (*19*). Conversely, a different study indicated putative transmission, finding RT-QuIC activity in 77.7% of CWD-inoculated *tg650* mice (6-fold overexpression of human PrP); 44.4% displayed progressive clinical signs (myoclonus) although only 1 mouse had histochemical abnormalities (*20*). Any potential transmission is cause for concern; thus, models that more closely represent humans are required.

The need for models that are more closely related to humans has been partially addressed using nonhuman primates. Transmission of CWD to squirrel monkeys has been readily demonstrated but, to date, transmission studies in cynomolgus macaques (a closer laboratory animal model to humans than squirrel monkeys) have not shown evidence of prion disease (*21*,*22*). In those studies, no markers of prion infection were found in macaques euthanized as many as 13 years after inoculation with CWD (*22*). In contrast, BSE readily transmitted to macaques, causing behavioral and cerebellar signs and progressing to extremely severe ataxia within a few weeks of initial clinical signs (*23*). For adult animals, euthanasia was required at ≈160 weeks after inoculation. This encouraging difference between BSE and CWD supports a strong species barrier against CWD infecting humans.

To further address those questions of susceptibility, we used human cerebral organoids (hCOs) to model CWD infection in human neural tissue. hCOs are spheres of self-structuring brain tissue grown in a dish and are the closest model to human brain currently available. They are susceptible to infection with human prions and faithfully propagate the infecting prion strain (*24*,*25*). Using the predominant codon 129 genotypes, including the most susceptible to BSE (129MM) and the most common codon 129 genotype in many countries (129MV), we sought to determine whether CWD infection could be established in human cerebral organoid cultures by direct exposure to high titers of CWD prions.

## Methods

### Human-Induced Pluripotent Stem Cells and Culture

The production and routine maintenance of the human-induced pluripotent stem cells (hu-iPSCs) used in this study have been described in detail previously (*25*). In brief, codon 129MV (ACS-1023; ATCC) and 129MM hu-iPSCs (RAH019A) (*26*) were routinely cultured on low growth factor Matrigel in mTeSR1 Plus medium with 5% CO_2_ in a humidified incubator and passaged before colonies started to contact each other.

### CRISPR/Cas9 Knockout of PRNP

Knockout (KO) of PRNP by CRISPR/Cas9 cloning was performed by Applied StemCell (https://www.appliedstemcell.com). The guide RNAs G1 GCTTCGGGCGCTTCTTGCAG and G2 CTGGGGGCAGCCGATACCCG were used to introduce a frameshift mutation around aa 21 (within the N terminal signal sequence) (Appendix).

### Human Cerebral Organoid Generation and Routine Culture

We generated cerebral organoids using the cerebral organoid differentiation kit (StemCell Technologies, https://www.stemcell.com), which follows the protocol described in Lancaster et al. (*27*). After differentiation, cultures were maintained in conical flasks on an orbital shaker at 80 rpm in complete maintenance medium: 1 × glutamax, 1 × penicillin/streptomycin solution, 0.5% vol/vol N2, 1% vol/vol B12 with retinoic acid (all ThermoFisher Scientific, https://www.thermofisher.com) and 0.5 × nonessential amino acids, 0.025% vol/vol insulin, and 0.00035% vol/vol 2-Merceptoethanol (all Sigma-Aldrich, https://www.sigmaaldrich.com) in 1:1 Neurobasal:DME-F12 medium (ThermoFisher Scientific), under standard incubator conditions (5% CO_2_, 37°C, humidified).

### Prion Infections of Human Cerebral Organoids

We cultured hCOs for 5 months before infection to enable the development of astrocytes and maturation of neurons (*28*). We diluted previously characterized brain homogenates (*29*) from an uninfected deer, a pool of 6 CWD-infected mule deer, a pool of 7 CWD-infected white-tailed deer, an uninfected elk, a pool of 6 CWD-infected elk, and sporadic CJD (sCJD) (MV2) into organoid maintenance media to a final concentration of 0.1% (tissue wet) wt/vol (Table). Control sCJD brain homogenate was a kind gift from Gianluigi Zanusso (University of Verona, Italy). At the start of infection, existing media were removed from the organoids and replaced with the inoculated media. At 24 hours after inoculation, we added an equivalent volume of fresh media to the cultures (diluting the original inoculum 1:1). We performed a full media and vessel exchange 7 days after initial exposure.

**Table:** Inoculum details used in the study of attempted CWD prion transmission to human cerebral organoids*

Sample	Name	log LD_50_/mg brain†	log SD_50_/mg brain‡
Human normal brain homogenate	hNBH	Negative	Negative
Human sporadic CJD MV2	CJD	ND	6.9
Deer normal brain homogenate	dNBH	Negative	Negative
Whitetail deer CWD§ (pool of 7)	dCWD1	5.6 (WTD)	6
Mule deer CWD¶ (pool of 6)	dCWD2	5.7 (MD)	6.2
Elk normal brain homogenate	eNBH	Negative	Negative
Elk CWD# (pool of 6)	eCWD	5.3	6.5

*CJD, Creutzfeldt-Jakob disease; CWD, chronic wasting disease; LD_50_, 50% lethal dose; negative, negative control; ND, not done; SD_50_, 50% seeding dose. †Adapted from Race et al. (*29*). ‡Inocula. §WTD-1 in nomenclature as found in (*29*). ¶MD-1 in nomenclature as found in (*29*) #Elk-2 in nomenclature as found in (*29*).

### RT-QuIC Analysis

We performed RT-QuIC assays as previously described (*25*). We homogenized organoids to 10% wt/vol in phosphate-buffered saline by motorized pestle and cleared by centrifugation at 2,000 × *g* for 2 minutes. We serially diluted organoid homogenates diluted in 0.1% sodium dodecyl sulfate/phosphate-buffered saline/N2 solution to 0.1% wt/vol, a 10^−3^ dilution, and loaded 1 μL into each well of a black 384-well plate with a clear bottom (Nunc) containing 49 μL of reaction mixture. RT-QuIC reaction mix contained 10 mM phosphate buffer (pH 7.4), 300 mM NaCl, 0.1 mg/ml of truncated hamster recombinant PrP (amino acids 90–231), 10 μM thioflavin T (ThT), 0.002% SDS (contributed by homogenate dilution), and 1 mM ethylenediaminetetraacetic acid tetrasodium salt (EDTA). We sealed plates using a Nalgene Nunc International sealer (ThermoFisher Scientific) and incubated in a FLUOstar Omega plate reader (BMG LabTech, https://www.bmglabtech.com) at 50°C with cycles of 60 seconds of shaking (700 rpm, double-orbital) and 60 seconds of rest throughout the 50-hour incubation time. We took ThT fluorescence measurements (excitation, 450 + 10 nm; emission, 480 + 10 nm [bottom read]) every 45 minutes. We ran quadruplicate reactions for each sample. We considered an individual reaction positive if its maximum fluorescence reading within 50 hours was >10% of the maximum fluorescence reading in the experiment. We considered a sample positive if >25% of the replicate reactions were scored as positive. We performed estimates of the concentrations of seeding activity using endpoint dilution analysis and calculated with Spearman-Kärber analyses as previously described (*25*) and provided as 50% seeding dose.

### Immunohistochemistry

We submitted 2–6 organoids from each experimental group for histologic studies. Organoids were immersed in 3.7% neutral buffered formalin for ≈24 hours before standard embedding in paraffin. We performed immunohistochemical (IHC) staining specifically for PrP using 3 different PrP antibodies: SAF32 (Cayman Chemical, https://www.caymanchem.com) (*30*), F89/160.1.5 (F89) (GeneTex, https://www.genetex.com) (*31*) and F99/97.6.1 (F99) (VMRD, Inc., https://vmrd.com) (*31*). We sectioned organoids into 5-µm slices and performed deparaffinization, antigen retrieval and staining using the Discovery Ultra Staining Module (Roche, https://www.roche.com). We retrieved antigens for all PrP IHC staining by using extended cell conditioning with CC1 buffer (Roche) containing Tris-Borate-EDTA, pH 8.0 for 100 minutes at 95°C. Before staining, we applied a horse serum blocker (Vector #136021) at 37°C for 20 minutes. To stain PrP, we applied either SAF32 at a dilution of 1:2,000, F89 at 1:250, or F99 at 1:25 for 1 hour at 37°C. We performed all antibody dilutions using antibody dilution buffer (Roche). The secondary antibody for all 3 primary PrP antibodies was horse anti-mouse IgG (Vector #30129), applied undiluted for 32 minutes at 37°C, followed by detection with ChromoMap DAB (Roche). We digitized and analyzed all histopathology slides using Aperio Imagescope software (https://www.leicabiosystems.com/us/digital-pathology/manage/aperio-imagescope).

### Proteinase K Digests and Western Blot Analysis

We performed proteinase K digests and Western blot analysis as described previously (*25*). In brief, we treated 10% organoid homogenates with 5 μg/mL proteinase K in 1% Sarkosyl for 1 hour at 37°C with 400 rpm shaking. We ran equal volumes of the digested 10% homogenates on Bolt 4%–12% Bis-Tris gels and transferred to PVDF membranes using the iBlot 2 transfer system (ThermoFisher Scientific). We detected PrP by using the 3F4 antibody (Millipore Sigma, https://www.emdmillipore.com) at a 1:10,000 dilution, visualized using ECL Select (Cytiva, https://www.cytivalifesciences.com), and imaged on the iBright imaging system (ThermoFisher Scientific). We visualized total protein with Coomassie blue staining.

### PrestoBlue and Lactate Dehydrogenase

We assessed relative organoid health using PrestoBlue metabolism and lactate dehydrogenase (LDH) release assays, per the manufacturer’s instructions. In brief, we plated 3–6 random representative organoids from each group in 24-well plates with 0.5 mL of fresh media. Approximately 24 hours later, we mixed 50 µL media with 50 µL LDH catalyst and dye in a 96-well plate, then incubated the plate for 15 minutes at 37°C before adding 25 µL of stop solution. We measured absorbance on the ClarioStar plate reader (BMG LabTech) at 460 nm, subtracting reference wavelength 690 nm from the reading. Once LDH was measured, we used the same organoids for PrestoBlue metabolism. We diluted PrestoBlue reagent in organoid maintenance media at a 1:10 ratio. We then removed the organoids’ existing media, added 0.5 mL of the PrestoBlue media, and incubated at 37°C for 30 minutes before transferring the media to a black 96-well plate for analysis. We measured fluorescence at 560 nm excitation and 590 nm emission in the ClarioStar plate reader. Values are presented as relative change from the normal brain homogenate (NBH) controls.

## Results

### Organoid Viability after CWD Exposure

**Figure 1:**
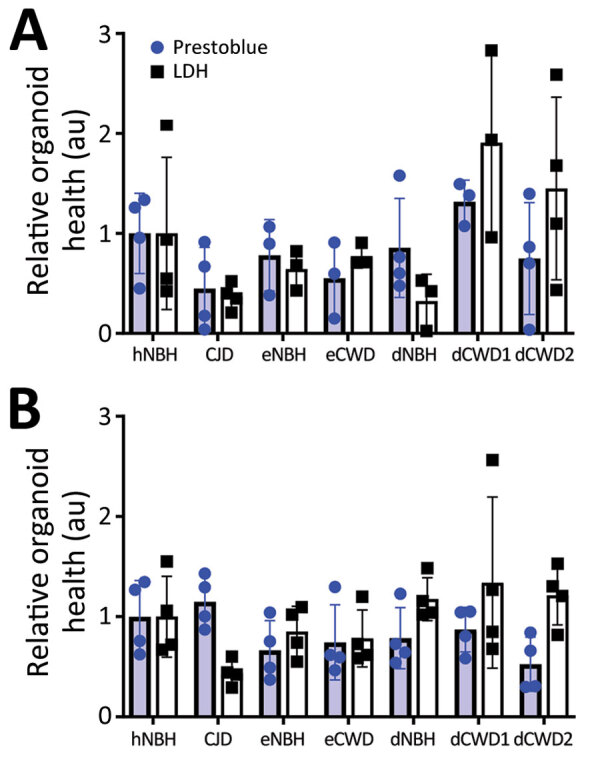
PrestoBlue viability and LDH release assays for the 129MM (A) and 129MV (B) representative organoids measured before harvest at 180 days post innoculation in study of lack of transmission of CWD prions to human cerebral organoids. Results indicate CWD exposure does not reduce organoid viability. Individual dots represent a single organoid, bars indicate the mean response, and error bars show SDs. No condition was statistically changed from controls as determined by 1-way analysis of variance with Welch’s correction. au, arbitrary units; CJD, Creutzfeldt-Jakob disease; CWD, chronic wasting disease; dCWD1, whitetail deer CWD; dCWD2, mule deer CWD; dNBH, deer normal brain homogenate; eCWD, elk CWD; eNBH, elk normal brain homogenate; hNBH, human normal brain homogenate; LDH, lactate dehydrogenase.

We immersed hCOs for 7 days in media containing negative control normal brain inocula either from humans (hNBH), deer (dNBH), or elk (eNBH) or infectious prions from sCJD human brain homogenate (CJD; positive control), 2 species of deer (dCWD1, dCWD2), or elk (eCWD), as previously described (Table). We monitored hCOs for 180 days after infection for visible signs of distress, including changes in appearance and metabolizing of the media. Before the final harvest, we assessed 3 or 4 organoids from each treatment group for differences in metabolism by PrestoBlue viability assay or cellular integrity by LDH release. Although there was wide variability in the organoid responses, no signs of decreased health were evident in any condition (Figure 1). Thus, exposure to the homogenates had no influence on organoid viability.

### Propagation of MV2 sCJD Prions in 129MM and 129MV Organoids

**Figure 2:**
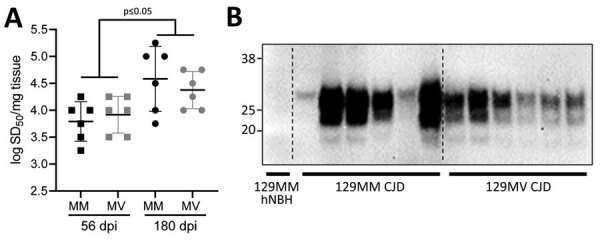
Demonstration of infection and accumulation of MV2 sporadic CJD prions in human cerebral organoids in study of lack of transmission of chronic wasting disease prions to human cerebral organoids*.* Both the 129MM and MV organoids were infected with MV2 sporadic CJD prions to ensure uptake and accumulation could be measured in both lines. A) Real-time quaking-induced conversion seeding activity; B) accumulation of protease resistant prion protein were assayed at 56 and 180 dpi. Each marker in panel A represents the organoid from an individual with the means and SDs of all organoids per condition indicated. Panel B indicates Western blots using prion 3F4 antibody following protease digest of lysates from 2 representative MM and 2 representative MV organoids that received the same starting inoculum (MV2 CJD) along with a 129MM 180dpi organoid that received hNBH. CJD, Creutzfeldt-Jakob disease; dpi, days postinoculation; hNBH, human normal brain homogenate.

We have previously demonstrated that 129MV hCOs are susceptible to infection with human 129MV prions (*25*,*32*) and that 129MM hCOs are susceptible to infection with human 129MM prions (*33*). As a positive control, and to ensure that 129MM organoids were also susceptible to 129MV prions, we inoculated both organoid lines (129MV and 129MM) with 129MV2 human sCJD prions and assessed RT-QuIC seeding activity and protease-resistant PrP (a biochemical marker of disease associated prion deposition) at 56 and 180 days postinoculation (dpi). RT-QuIC seeding activity was present at 56 dpi and showed a significant increase after incubation to 180 dpi (Figure 2, panel A). No protease-resistant PrP could be detected within the organoids at 56 dpi, but significant accumulation had occurred by 180 dpi (Figure 2, panel B). Therefore, the CJD control organoids did take up infection and propagate it, with accumulation over time.

### No Propagation of PrP Seeding Activity in CWD-Exposed Organoids

**Figure 3:**
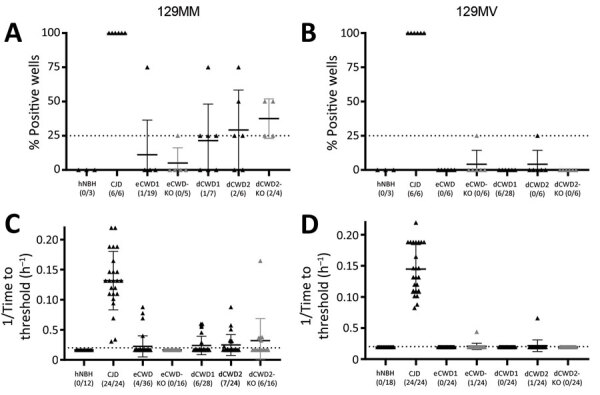
Real-time quaking-induced conversion (RT-QuIC) seeding activity of CWD-exposed organoids in study of lack of transmission of chronic wasting disease prions to human cerebral organoids*.* RT-QuIC seeding activity in organoids harvested at 180 days postinoculation is shown as either % positive wells (A, B) or the reciprocal of time-to-significance threshold (C, D) for the 129MM (A, C) and 129MV (B, D) organoids. Dotted lines indicate the threshold above which a sample would be classified as positive. Individual dots show single organoids with the means and SDs indicated. Gray symbols are indicative of knockout organoids. No significant differences were observed between the CWD-inoculated wild type organoids and their corresponding knockout organoids by Welch’s t-test. CJD, Creutzfeldt-Jakob disease; CWD, chronic wasting disease; dCWD1, whitetail deer CWD; dCWD2, mule deer CWD; dNBH, deer normal brain homogenate; eCWD, elk CWD; eNBH, elk normal brain homogenate; hNBH, human normal brain homogenate.

RT-QuIC analysis of the organoids collected at 180 dpi showed that, with the exception of the CJD-positive controls, no inoculum in either the codon 129MM or 129MV organoids resulted in the production of significant seeding activity (Figure 3). In the case of the 129MM hCOs, some weak positive signals were observed (Figure 3; Appendix Figure 2). However, similar observations were made in genetically matched PrP KO organoids (Figure 3; Appendix Figure 2, gray markers), which have no PrP substrate for propagation of misfolding. Coupled with a decline in seeding activity over the course of the experiment (Appendix Figure 2), this finding suggests that the observed signals are a likely result of residual inocula persisting in the organoids for a prolonged period, producing false-positive reactions. Altogether, RT-QuIC analysis for prion seeding activity indicated that none of the CWD-inoculated organoids contained seeding activity indicative of actively propagating infection, such as is seen with CJD-infected hCOs.

### No Protease-Resistant PrP in CWD-Exposed Organoids

**Figure 4:**
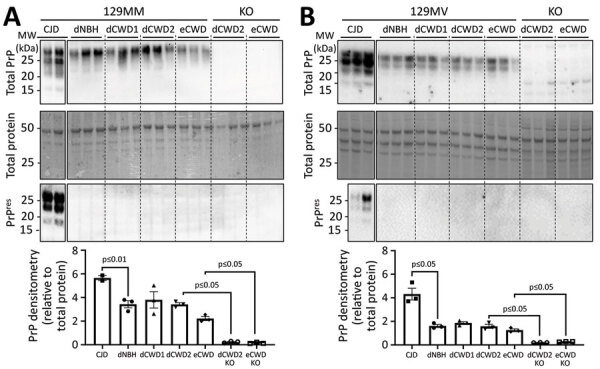
Western blot analysis of total PrP and PrP^res^ levels in representative organoids for sporadic CJD and CWD-exposed human cerebral organoids in study of lack of transmission of chronic wasting disease prions to 129MM (A) and 129MV (B) human cerebral organoids. Matched KO organoids inoculated with dCWD2 or eCWD are shown for comparison. Densitometric analysis (shown in bottom panels) shows total PrP levels relative to total protein with each point representing an individual organoid; means and SDs are indicated. p values were calculated using Welch’s t-test. Uncropped Western analyses are shown in Appendix Figure 3. CJD, Creutzfeldt-Jakob disease; CWD, chronic wasting disease; dCWD1, whitetail deer CWD; dCWD2, mule deer CWD; dNBH, deer normal brain homogenate; eCWD, elk CWD; eNBH, elk normal brain homogenate; hNBH, human normal brain homogenate; KO, knockout; PrP, prion protein; PrP^res^, protease-resistant prion protein.

Accumulation of disease-associated, protease-resistant PrP is also a hallmark of prion disease. Therefore, we probed organoid homogenates from the CWD infections for PrP levels with and without proteinase K digestion. Protease-resistant PrP was only observed in the CJD-infected control organoids and not in any of the CWD-exposed organoids (Figure 4), and none of the CWD conditions showed a significant increase in total PrP levels (Figure 4).

### No Human Prion Deposition in CWD-Exposed Organoids

**Figure 5:**
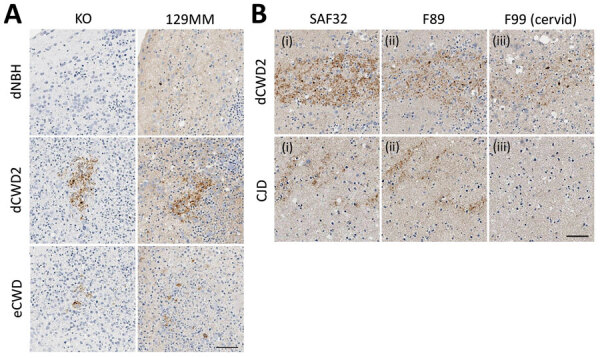
Prion protein (PrP) deposits in organoids. A) Deposits of PrP in 129MM and KO organoids detected with SAF32 antibody in study of lack of transmission of chronic wasting disease prions to human cerebral organoids. B) Prion deposition in sequential slices of representative 129MV organoids is detected by both SAF32 (i) and F89 (ii) total PrP antibodies in both CWD and CJD inoculated organoids. Cervid PrP-specific antibody F99 (iii) detects the same deposits in the just the CWD inoculated organoids, indicating that the deposits are of cervid origin (i.e., inocula) and not misfolded human PrP. Scale bars indicate 50 µm. CJD, Creutzfeldt-Jakob disease; CWD, chronic wasting disease; dCWD2, mule deer CWD; dNBH, deer normal brain homogenate; eCWD, elk CWD; KO, knockout.

Histologic examination of the tissues at the conclusion of the experiment (180 dpi) showed no evidence of pathology or plaques within the CWD-inoculated organoids. However, several dCWD2 and eCWD organoids contained scattered regions of abnormal PrP deposition. Although the PrP KO organoids showed no native PrP staining compared with the background hue of normal PrP expression seen in the wild type organoids, further examination revealed abnormal PrP deposits similar to those seen in the wild type organoids using the SAF32 PrP antibody. This finding indicates that the deposits are likely residual CWD inoculum and not de novo deposition of human PrP (Figure 5, panel A). To verify that those deposits were indeed residual inocula, we further analyzed the tissue slices using F89 and F99 PrP antibodies. F89 is a PrP antibody that detects both cervid and human PrP, similar to SAF32, whereas F99 is a PrP antibody that detects only cervid PrP and not human PrP. Staining with F89 demonstrated similar results for both CJD- and CWD-exposed organoids, similar to that of SAF32. However, when stained with the F99 cervid PrP antibody, only the CWD-exposed hCOs showed PrP staining, confirming that the positive staining material was cervid in origin and not converted human PrP (Figure 5, panel B; Appendix Figure 4). Altogether, despite clear accumulation of pathogenic (seeding positive, protease-resistant) PrP in the sCJD-infected organoids, prolonged, high-dose exposure of hCOs to CWD prions was not sufficient to cause conversion of human PrP or disease propagation.

## Discussion

The data presented in this study show that, despite weeklong exposure to CWD prions with high infectivity and the capacity to readily become infected with CJD prions, hCOs were not capable of propagating CWD prions. This finding indicates that, even after direct exposure of human central nervous system tissues to CWD prions, a substantial resistance or barrier to the propagation of infection exists.

Although we tested the 2 most common PrP genotypes (129MM and 129MV), our results do not preclude the possibility that homozygosity for the valine allele at codon 129 would result in increased susceptibility to CWD. There is precedent for that possibility. Wang et al. (*34*) were able to generate human CWD prions by using the protein misfolding cyclic amplification (PMCA) assay. Using that approach, the authors found that elk CWD prions could trigger conversion and amplification of human 129VV PrP in brain homogenate that was subsequently transmissible to mice. Conversely, 129MM human brain homogenate would not amplify CWD, and 129 MV brain homogenate was not tested. Other studies, however, have demonstrated CWD codon 129 susceptibility similar to BSE in vitro, where methionine homozygosity shows greater susceptibility to conversion with some CWD samples (*11*). Barria et al. (*11*) found that humanized mouse-derived MM substrates showed some degree of conversion by white-tailed deer CWD prions by PMCA, whereas MV and VV substrates were resistant. All 3 genotypes, however, showed susceptibility to reindeer CWD prions. Those assays showed that, given the right circumstances, human PrP can be seeded by cervid CWD prions; however, they forced a reaction in a way that might not be representative of the genuine risk to humans from a more natural exposure. The lack of propagation in the organoid model supports the idea that other aspects of the PMCA reaction, such as the radicals formed by sonolysis (*35*), might be necessary to initiate the observed conversion.

A lack of transmission of CWD to the human cerebral organoid model supports the data found in macaques, where transmission did not produce prion disease (*21*,*22*,*29*). That finding is in clear contrast to BSE, in which the macaques infected with BSE prions succumbed to prion disease (*36*,*37*). BSE has also been demonstrated to infect humanized mice (*38*,*39*). Inoculation of humanized mice with CWD has been mostly unsuccessful in causing infection (*13*,*17*–*19*), but transmission of CWD to humanized mice was observed in 1 study (*20*). Those mice overexpressed (≈6-fold) human PrP with methionine at codon 129. Our cerebral organoids, which also express methionine at codon 129 (129MM and 129MV), are a model of completely human brain tissue with normal PrP expression levels. Thus, this finding suggests that the mouse background, possibly in combination with overexpression of human PrP, is a more favorable environment than human brain tissue for CWD infection to occur.

The organoid model, although the closest to human brain tissue currently available, has various limitations and does not reproduce all aspects of the human brain (*40*). Thus, hCOs might be lacking factors or cell types that would make the human brain more susceptible to CWD prions. Many more unknowns cannot be accounted for in this system. For example, we cannot exclude unknown susceptibility factors that could make a small population more vulnerable to infection, and we have not tested all cervid genotypes against all human genotypes. Likewise, the possibility remains that new strains of CWD with the capacity to cross the species barrier could emerge in the future. For now, our data suggest that such seeding of human PrP by cervid CWD prions is unlikely to occur or be sustained in human brain tissue.

In conclusion, experimental transmissions of 3 sources of CWD to 2 *PRNP* codon 129 genotypes of human cerebral organoids were unsuccessful. Although we cannot rule out the possibility of CWD crossing into humans, our data suggest that a significant species barrier exists, even when human brain tissue is directly exposed to high-titer CWD brain homogenate for a prolonged period.

Appendix Additional information about lack of transmission of chronic wasting disease prions to human cerebral organoids
