# Supplementary material for: Lack of Transmission of Chronic Wasting Disease Prions to Human Cerebral Organoids
Source: Emerg Infect Dis. 2024 Jun;30(6):1193–202. doi: 10.3201/eid3006.231568 (PMC11138967; doi:10.3201/eid3006.231568)
Supplement: Appendix — Additional information about lack of transmission of chronic wasting disease prions to human cerebral organoids [file 23-1568-Techapp-s1.pdf]

Article DOI: https://doi.org/10.3201/eid3006.231568

EID cannot ensure accessibility for supplementary materials supplied by authors. Readers who have difficulty accessing supplementary content should contact the authors for assistance.

Lack of Transmission of Chronic Wasting Disease Prions to Human Cerebral Organoids

Appendix

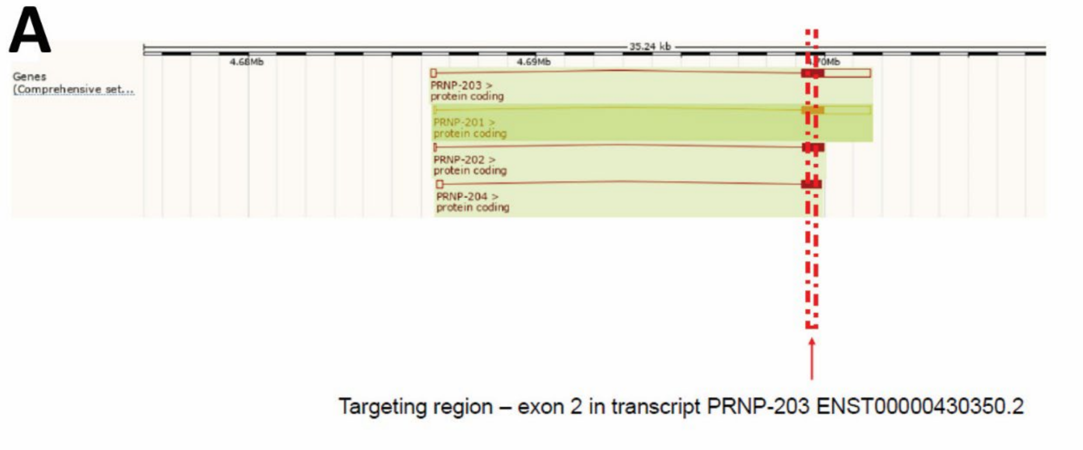

B

The activity and specificity of gRNA for *PRNP*

	Sequence	PAM	Exon	Activity	Off-target
C2013-g1	GCTTCGGGCGCTTCTTGCAG	AGG	2	51	89

Guide Details	
Cut site	
GC %	65
Activity	51
Off-target	89

OFF-TARGET ANALYSIS					
Mismatches	0	1	2	3	Total
Coding	0	0	0	0	0
Non-coding	0	0	0	2	2

	Sequence	PAM	Exon	Activity	Off-target
C2013-g2	CTGGGGGCAGCCGATACCCG	GGG	2	61	93

Guide Details	
Cut site	
GC %	75
Activity	61
Off-target	93

OFF-TARGET ANALYSIS					
Mismatches	0	1	2	3	Total
Coding	0	0	0	0	0
Non-coding	0	0	0	4	4

C

gRNA profiles at the locus (*PRNP*)

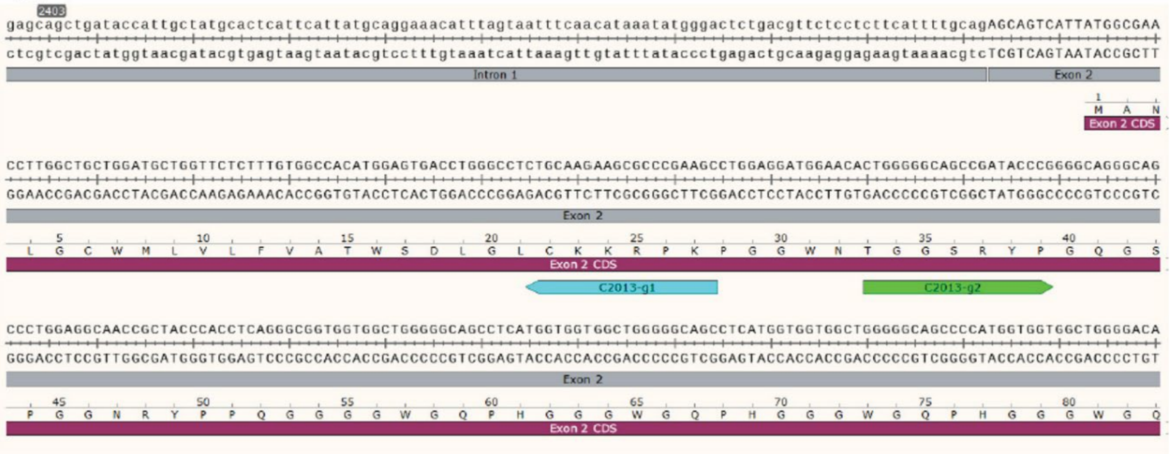

D

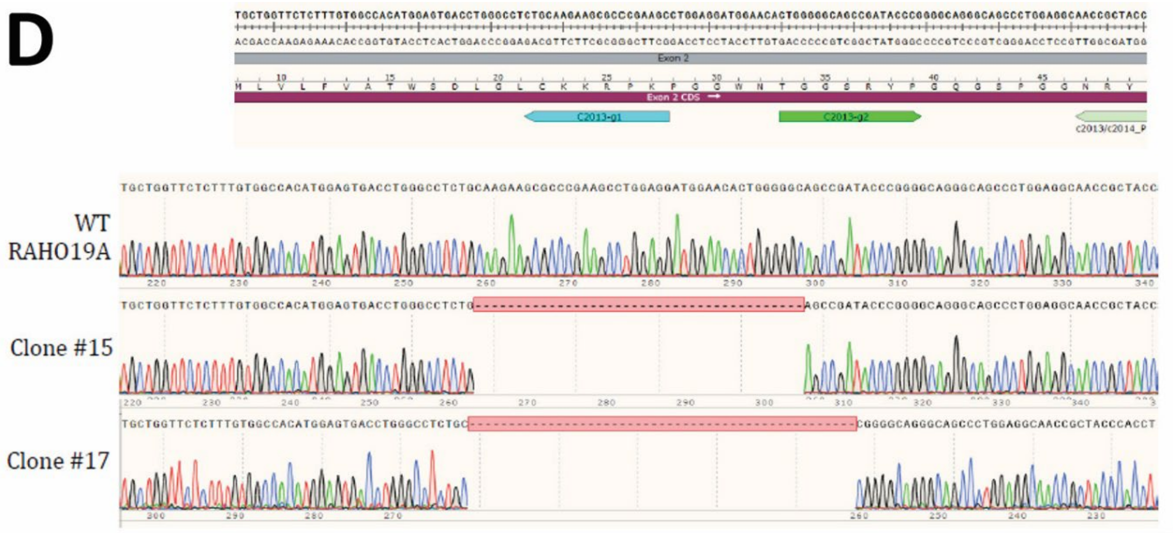

E

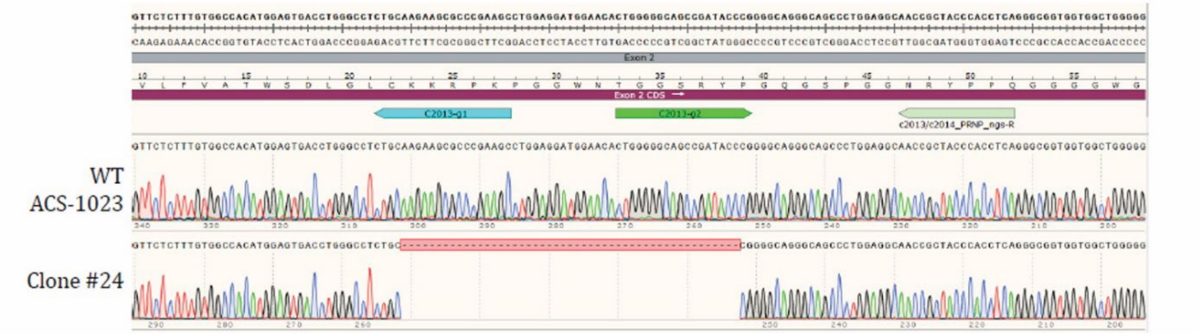

Appendix Figure 1. Generation of PRNP KO iPSCs. **A.** *PRNP* region targeted for frameshift mutation. **B.** gRNA design, activity and off-target activity and mismatch. **C.** gRNA locations with the prion protein coding region of exon 2. Sequencing of the clones from the 129MM (**D**) and 129M/V (**E**) iPSC lines.

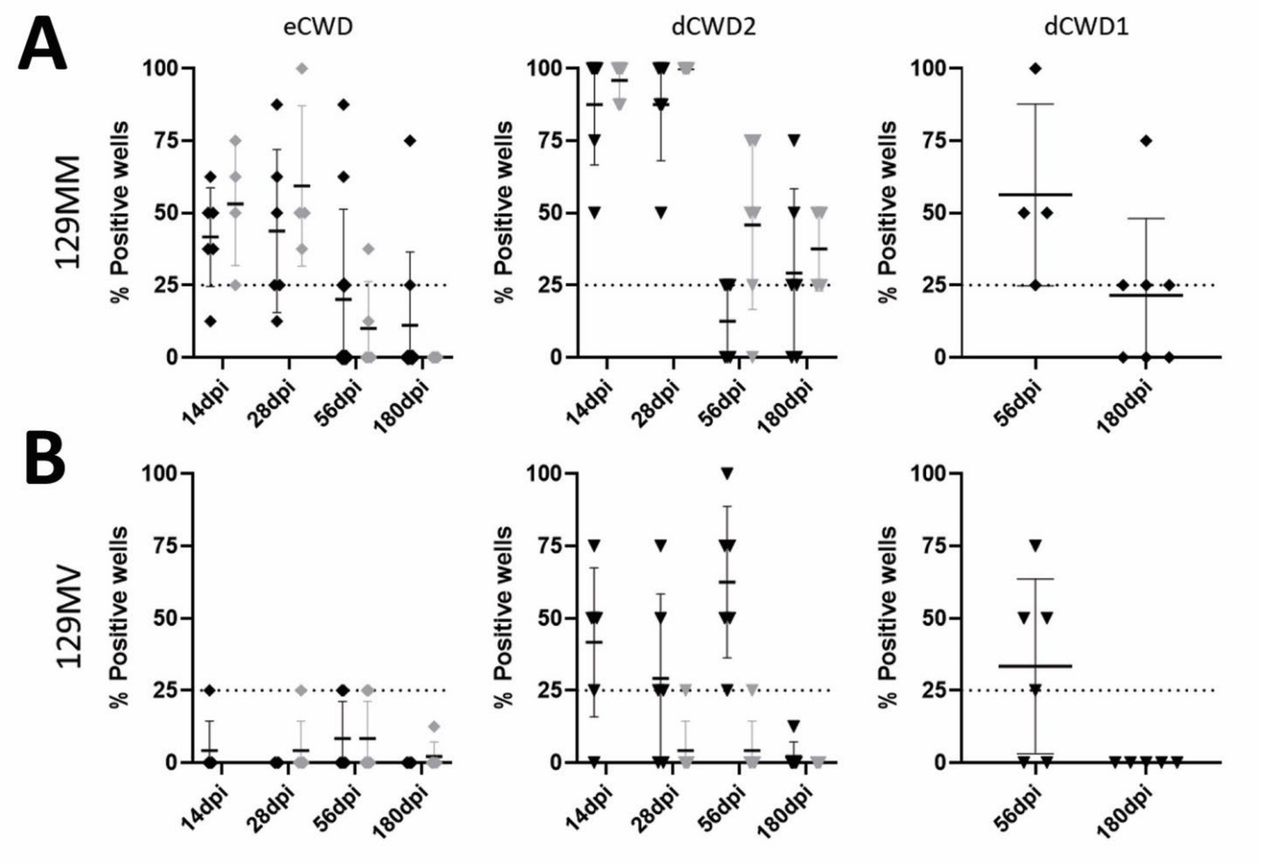

Appendix Figure 2. Loss of input seeding activity over time. RT-QuIC positive wells showing the decline of the residual inoculum over time. Dots show the percentage positive wells of a single organoid with WT shown in black and PRNP knockout in gray, bars show the mean and standard deviation.

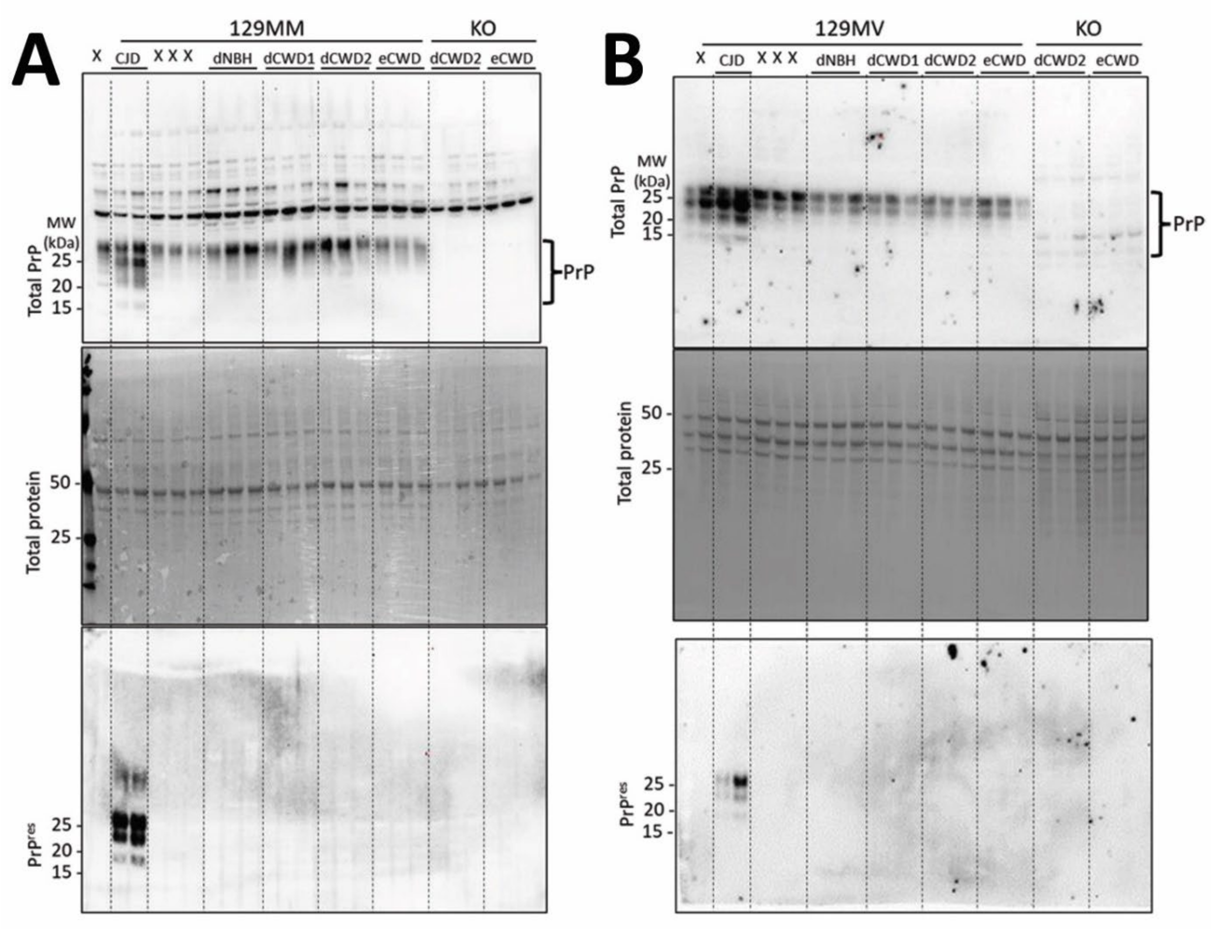

Appendix Figure 3. Uncropped western blots. Uncropped, full sized western blots shown in Figure 4. “X” indicates lanes that were cropped from Figure 4 as they were not pertinent to this study.

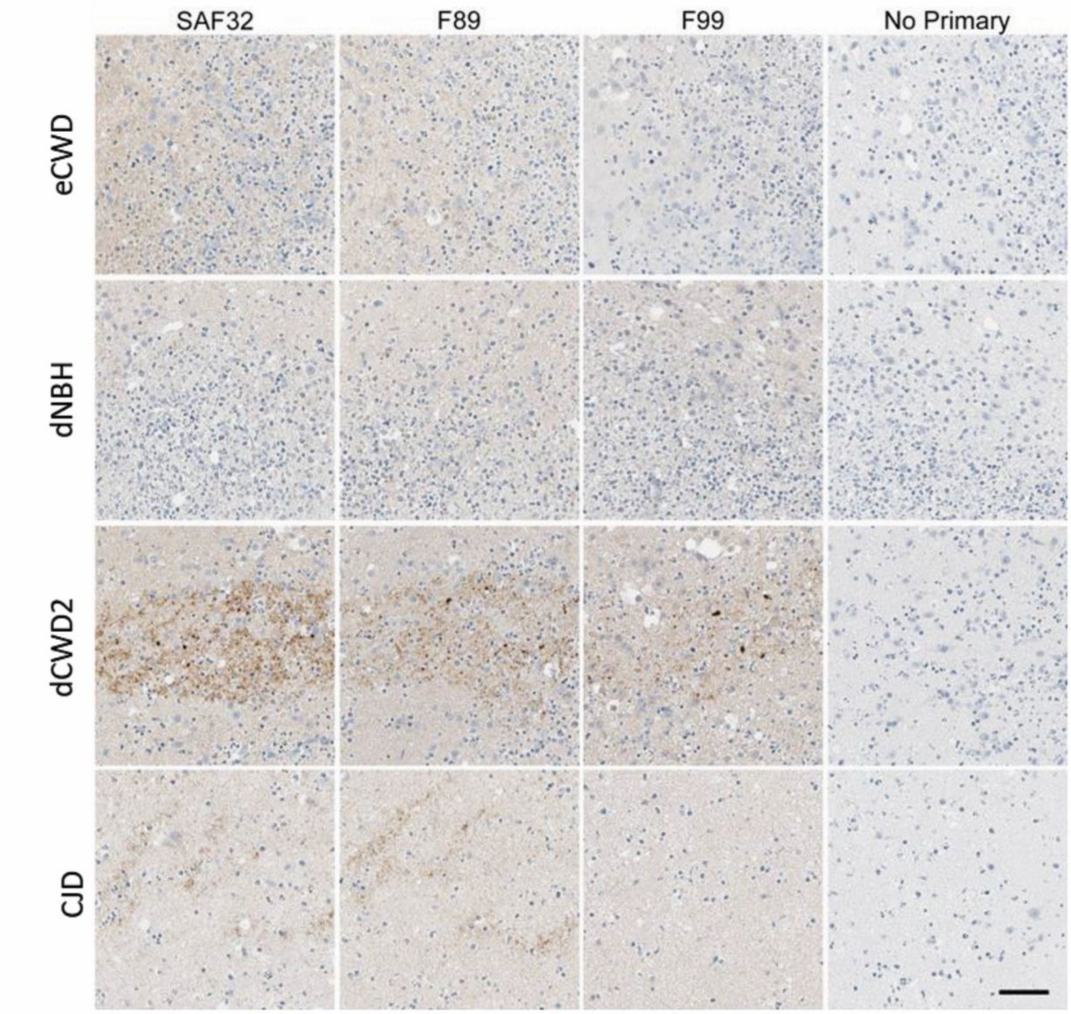

Appendix Figure 4. CWD inoculum persists in organoids. Immunohistochemistry staining for PrP (brown) using the antibodies indicated and showing that detected PrP deposits are residual cervid PrP.
